# Investigation of the Influence of Pine Cone and Pine Resin Addition on the Properties of Plaster Composites

**DOI:** 10.3390/polym18121501

**Published:** 2026-06-16

**Authors:** Ayse Bicer, Celal Kistak, Ali Taskiran, Nevin Celik

**Affiliations:** 1 Department of Bioengineering, Malatya Turgut Ozal University, Malatya 44210, Turkey; ayse.bicer@ozal.edu.tr; 2 Department of Mechanical Engineering, Firat University, Elazig 23119, Turkey; ckistak@firat.edu.tr; 3 Department of Mechanical Engineering, Sirnak University, Sirnak 73000, Turkey; taskiranalii@gmail.com

**Keywords:** pine tree resin, pine cone, gypsum plaster, thermal-mechanical properties, composite building materials

## Abstract

The physical characteristics of gypsum plaster composites made from agricultural wastes of pine cones (PCs) and pine resin (PR), as sustainable substitutes for traditional natural fillers, are examined in this work. Particle sizes of 3–5 mm, 0–3 mm, and powder were used to classify the PCs after grinding. First, gypsum plaster was replaced with PC particles at 20%, 40%, 60%, and 80% by weight for each group to produce resin-free specimens. To create artificial porosity and improve the matrix’s binding strength, 1% PR by total weight was then added to duplicate mixes, yielding 24 different sample combinations. In resin-free samples, the addition of 3–5 mm, 0–3 mm, and powder PC (in ratios ranging from 20% to 80%) reduced the thermal conductivities by 35.16%, 33.84%, and 34.97%, respectively, and the compressive strength values by 82.00%, 77.18%, and 77.50%. Further decreases in thermal conductivity (29.36%, 32.05%, and 33.66%) and compressive strength (76.89%, 75.64%, and 75.48%) were observed in PR-modified samples. The results show that adding PCs and PR to gypsum plaster considerably improves the plaster’s thermal insulation while marginally reducing its compressive strength loss.

## 1. Introduction

### 1.1. Using Plant-Based Materials as Aggregate

Plant particles, also known as “plant aggregates,” have been used as thermal-insulating building materials for approximately 30 years to address environmental concerns in the construction industry. They can be used in their bulk form or combined with mineral binders to create “plant-based concrete.” Many plant aggregates have already been tested with mineral binders, including hemp shiv, coconut coir, flax shiv, wood chip, cereal or oilseed straws, rice husk, corn cob, diss stem, bamboo stem, cane bagasse, sugar beet pulp, miscanthus stem, and lavender straw [1].

Using plant-based materials offers significant benefits. First of all, (i) it significantly reduces environmental impact. Cement production alone is a major contributor to CO_2_ emissions. When plant-based material ash is used as a partial replacement, the demand for cement decreases. This directly reduces greenhouse gas (GHG) emissions and helps address climate change. (ii) It addresses the growing waste-disposal problem. Plant-based wastes are often burned or dumped, causing pollution. Incorporating them into concrete gives these materials a second life [2]. (iii) Some plant-based materials enhance concrete properties (coconut or palm kernel shells are used to produce lightweight concrete) [3]. (iv) The addition of plant-based materials to concrete reduces construction costs, especially in regions where such materials are abundant.

The rapid advancement of green construction technology has led to several research studies on the properties of plant-based concrete in recent years. Sáez-Pérez et al. [4] recently examined the factors—such as aggregate quality, binder choice, constituent dose, and production methods—that affect the properties and performance of hemp aggregate concrete. Similarly, Jami et al. [5] conducted another review on the properties of hemp concrete. Interestingly, they discussed the typical properties of hemp concrete, its potential applications in the building sector, and the connections between mixing and production processes. Ratsimbazafy [6] presented a review of the multi-physical properties of plant aggregates and their effects on the properties of plant-based concretes. Bourbia et al. [7] investigated the use of date palm shives as a bio-based aggregate in lightweight insulating concrete, addressing the need for low-impact construction materials to reduce the building sector’s carbon footprint.

A promising sustainable alternative lies in plant-based waste, particularly abundant resources such as pine cones (PCs) and pine tree resin (PR). Millions of tons of PC particles are generated annually worldwide as agricultural and forestry by-products. Although the exact global yield is challenging to quantify, the volume is substantial. Improper disposal of these wastes—such as open burning or environmental abandonment—blocks sunlight from reaching the soil, attracts pests, and significantly exacerbates forest fire hazards. Despite being low-cost, renewable resource-rich in cellulose, hemicellulose, and lignin, the effective valorization of PCs remains a significant challenge.

A literature review reveals that PCs, despite being a renewable, inexpensive, lightweight, easily accessible, and environmentally friendly natural fiber resource that is often discarded as organic waste worldwide, are not being adequately utilized. Research on the use of PCPs in concrete and plaster is limited. This research can be divided into two groups. The first group consists of studies on the use of PCPs as aggregates, summarized below. Kistak et al. [8] previously investigated the thermophysical properties of PR-modified concrete and cement with PC aggregates and suggested that these materials could be used as insulation in buildings. Bayraktar et al. [9] produced alkali-activated foamed concrete using fly ash and PC powder. The effects of the amounts of PC powder and fly ash on density, porosity, water absorption, thermal conductivity, absorbency, and compressive strength were evaluated. Singh et al. [10] investigated the mechanical and thermophysical properties of concretes using PCs as coarse aggregate. It was found that the compressive, tensile, flexural, and modulus of elasticity strengths of PC concrete were 34.98%, 30.23%, 24.94%, and 33.78% lower, respectively, than those of concrete without PCs. Arrakhiz et al. [11] used PC fibers to reinforce thermoplastic composites, demonstrating excellent mechanical and thermal properties. Agayev and Ozdemir [12] used powdered PCs in concrete to improve mechanical and thermal properties. Additional studies by Efe [13] and Basturk et al. [14] confirmed that the addition of PCs increased stiffness in biocomposites and epoxy resin composites. Ayrilmis et al. [15] used PC powder in medium-density fiberboard and found that formaldehyde emissions and water resistance were improved.

### 1.2. Using Gypsum as Binder

The choice of binder was influenced by the requirement to produce building materials with low energy use, low CO_2_ emissions, and high utilization of waste materials. As a result, the first requirement was to replace the cement binder with a less energy-intensive binder that was based on conventional building techniques in the area. Using local materials to avoid transportation-related increases in the final product’s carbon footprint was the second criterion [16]. Bibliographic research [17] has led us to integrate gypsum into binder recipes.

Plant-based materials have been widely explored in gypsum-bound concretes to reduce density, improve insulation, and increase sustainability. However, their interaction with gypsum (calcium sulfate hemihydrate) is quite different from Portland cement systems [18]. Uwizeyimana et al. [19] investigated the thermal recycling of gypsum–hemp bio-concrete, in which gypsum acts as the binder and hemp shiv as the aggregate. Physical, thermal, and mechanical properties were evaluated. Results showed that gypsum–hemp bio-concrete exhibits increased density (368 to 587 kg/m^3^) and compressive strength (0.05 to 0.52 MPa), accompanied by a moderate increase in thermal conductivity (0.081 to 0.096 W/mK). Goh et al. [20] used palm oil fuel ash and gypsum powder as partial replacements for cement in self-compacting concrete. The specimens were subjected to compressive strength tests using standardized testing machines. The results showed that increasing the gypsum proportion significantly reduced compressive strength, and the addition of palm oil fuel ash generally enhanced stiffness, whereas higher gypsum content had a reducing effect. Lalitsuda et al. [21] investigated the utilization of waste from power plants, construction and demolition, and agriculture by varying the ratios of flue-gas desulfurization gypsum, construction and demolition waste, and oil palm trunks in concrete production. All concrete brick specimens were tested for compressive strength and water absorption. It was reported that the compressive strength of concrete brick specimens and their water absorption were within acceptable limits for the standards.

### 1.3. Using Plant-Based Resin as Binder

The use of plant-based resin, together with porous aggregates, generally increases the insulation and strength properties of concrete. Because the resin absorbs water during soaking, swells slightly, and, when mixed with gypsum, loses this water as the sample dries, it forms artificial pores in the gypsum. Devecioglu and Bicer [22] added tragacanth resin and expanded clay to produce concretes with low thermal conductivity (e.g., 0.140 W/Mk). Bicer and Kar [23] reported favorable thermal and mechanical properties in concretes containing apricot resin and expanded polystyrene aggregates. Bicer [24] determined favorable thermal and mechanical properties by examining concretes containing PR resin and fly ash aggregates. McSwiggan et al. [25] evaluated a bio-based resin as a replacement for a standard epoxy in reinforced concrete and reported bond strength and durability comparable to those of the standard epoxy. Bicer and Celik [26] investigated the thermomechanical properties of gypsum plasters using natural resins.

### 1.4. Problem Statement

Although there have been some plant-based concretes as summarized in the literature survey, the PCs, which are a very common agricultural waste in the world, were rarely used to produce concretes. This study differs from similar studies by evaluating the use of waste PC aggregates and PR together to produce plant-based lightweight concrete. For this purpose, PC aggregates having sizes of 3–5 mm, 0–3 mm, and 0 mm (in powder form) were mixed with PR, gypsum plaster and water in various weight ratios. New concrete samples were produced with or without PR added to the concrete mixture. The new samples were then subjected to some important tests, such as density measurements, thermal conductivity tests, compressive strength tests, ultrasonic pulse velocity tests, and water absorption rate tests.

## 2. Materials and Methods

### 2.1. Materials

#### 2.1.1. Pine Cone (PC)

Pine trees belong to the Pinaceae family, which includes up to ten genera and 230 species distributed worldwide. A pine tree is a type of evergreen conifer in the genus Pinus. Pines are known for their needle-like leaves that stay green year-round. The tree’s seeds are called pine cones (PCs). The uses of PCs span from industrial applications to crafts and environmental solutions. PCs are not just forest waste—they are renewable, low-cost bio-resources with growing relevance in sustainable construction, energy, and environmental engineering [27].

**Figure 1:**
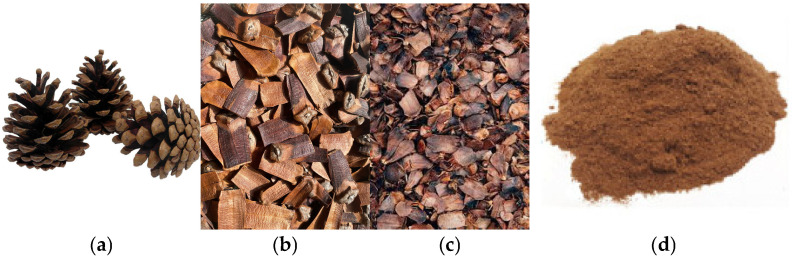
Form of PC: (**a**) raw PC, (**b**) crumbled PC, (**c**) crushed PC, (**d**) powder PC.

The PCs were collected on the Firat University campus, which has hundreds of pine trees. Before grinding, the PCs were left under the sun for 1 week to ensure moisture was removed. A simple hair dryer was used to blow air onto the PCs to remove the seeds, dirt, and dead insects/ants inside the PC’s leaf layers. First, the PCs were broken into smpair of garden shears. Then, a sharp knife was used to cut the pieces to the desired size. The first portion consisted of PC particles with diameters ranging from 3 to 5 mm that were coarsely crushed (Figure 1b). The coarse particles were further ground to a size range of 0–3 mm in order to obtain the second fraction (Figure 1c). PC that was finely ground made up the third portion (Figure 1d).

#### 2.1.2. Pine Tree Resin (PR)

Within the Pinaceae family, the Pinus genus stands out as the one with the highest investment in resin production. In pine trees, the resin is produced and accumulated at high concentrations (up to 10–20% of dry mass) in all tissues (stems, roots, branches, needles, and even cones). PR is used to seal wounds and protect against insects, fungi, and disease. It is made mainly of terpenes and resin acids and hardens when exposed to air.

PR is a fascinating natural material with a fairly complex chemical makeup and a very specific biological origin. PR is mainly composed of terpenes (volatile fraction) and resin acids (non-volatile fraction). The terpenes are low-molecular-weight hydrocarbons. They are responsible for the distinct pine smell and high volatility. This fraction is commonly distilled into turpentine. The main compound of resin acid is abietic acid. They have a higher molecular weight. They are sticky and solid after evaporation [27].

**Figure 2:**
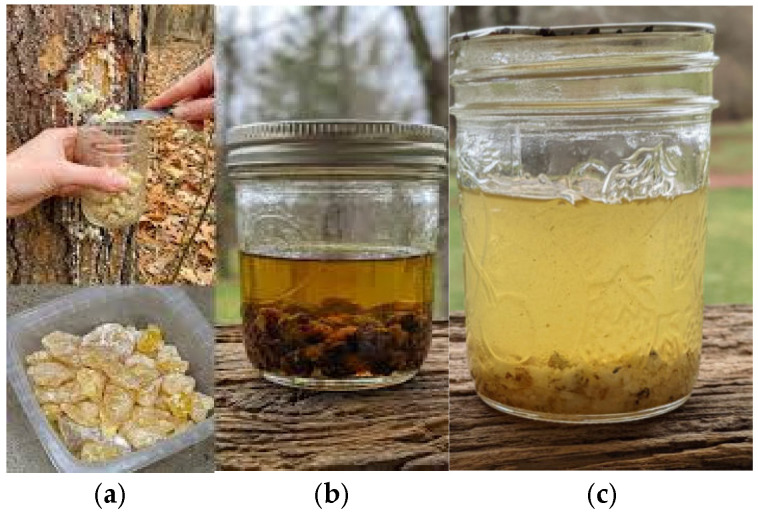
Preparing extract of resin: (**a**) collecting from the tree, (**b**) keeping in water, (**c**) extract form.

The PR is a naturally occurring exudate from pine bark that adheres firmly to the bark surface after solidifying via air oxidation (Figure 2a). The solid PR obtained for this investigation was milled into a fine powder (Figure 2b). The PR powder was then soaked in water for 48 h, after which it was combined with gypsum to produce an aqueous resin extract (Figure 2c).

The main goals of adding PR to the plaster matrix are (i) to increase the interfacial bond strength of the gypsum matrix during resin curing, and (ii) to create artificial pores within the microstructure, thereby improving thermal insulation.

#### 2.1.3. Gypsum

**Figure 3:**
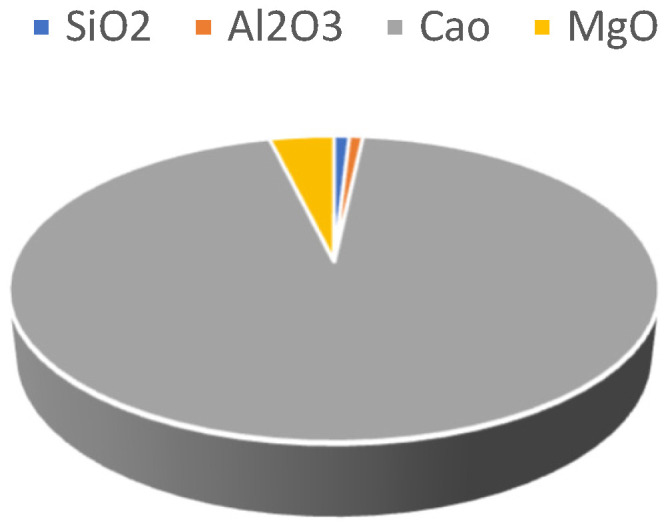
Composition of the gypsum.

Gypsum binders are inorganic, air-hardening mineral materials. Both natural and synthetic raw materials can be used for manufacturing binders. Their distinctive feature is the presence of hydrous calcium sulfate CaSO_4_·2H_2_O (gypsum) or anhydrous calcium sulfate CaSO4 (anhydrite). The chemical composition of the gypsum is given in Figure 3.

The gypsum used in the concrete samples is MAKONAT EKO brand powder form gypsum [28]. In gypsum–plant aggregate concretes, the β-hemihydrate (namely plaster of Paris) grade is most common due to its cost and workability. Hence, the gypsum used in the present study is β-hemihydrate. The properties of the gypsum are listed in Table 1.

### 2.2. Preparation of the Samples

When mixing the samples, two scaled containers showing volumetric values of 0.2, 0.4, 0.6, 0.8, and 1 L are used first. Volumetric ratios are determined using these containers. For example, if a PC is placed in 0.2 L of one container, gypsum is placed in 0.8 L of the other container. Then, the masses of PC and gypsum are determined by measuring them separately on a precision balance (like 90 g PC and 1520 g gypsum). Water equal to half the total mass of gypsum and PC is added to a large mixing container, since the water demand for the mixtures is decided by using the ratio of the following:

(1) $$\frac{water}{PC+gypsum}=0.5$$

Then, the three products are mixed by a mixer. Thus, a sample with a volumetric ratio of 20% PC is created.

For the samples including PR, the PR dissolved in water is fixed at 1% of the total mass of the gypsum–PC mixture. The masses of the samples are listed in Table 2.

Following mechanical mixing for approximately 3 min, the fresh mortars were cast into standard metal molds. Prismatic molds (20 mm × 60 mm × 150 mm) were utilized for thermal tests, while cubic molds (100 mm × 100 mm × 100 mm) were prepared for mechanical tests. The samples were demolded after 24 h and subsequently cured under ambient laboratory conditions for 28 days to ensure complete drying.

### 2.3. Measurement Methods

**Figure 4:**
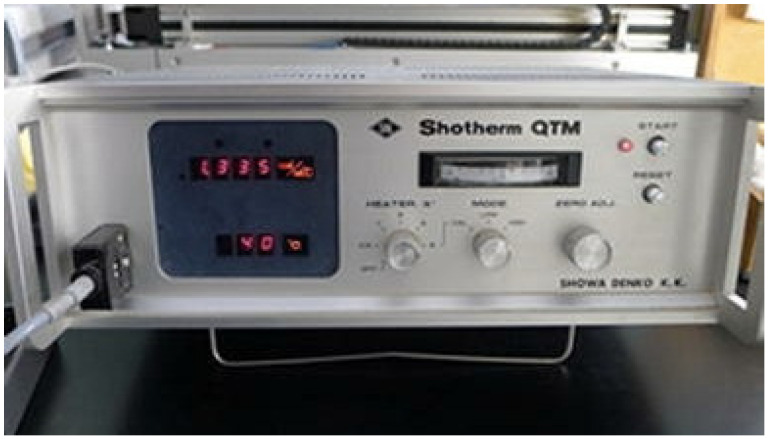
Thermal conductivity measuring device [29].

Thermal conductivity (*k*) measurements of the samples were performed with a “Shotherm-QTM” device that uses the hot-wire method and complies with DIN 51046 [29]. The Shotherm QTM (often referred to as the Kemtherm QTM series) is a rapid thermal conductivity testing device manufactured by Kyoto Electronics Manufacturing (KEM). A probe containing a single hot wire and a thermocouple is placed on the flat surface of the sample. The results can be obtained in 60 to 120 s, depending on the material, with a measuring range of 0.023 to ~11.63 W/mK (depending on the specific probe/model) and featuring a measuring precision of ±5% of the reading. It operates effectively in ambient environments, with some models handling temperatures from −10 °C to +200 °C. Figure 4 shows a photo of the device.

**Figure 5:**
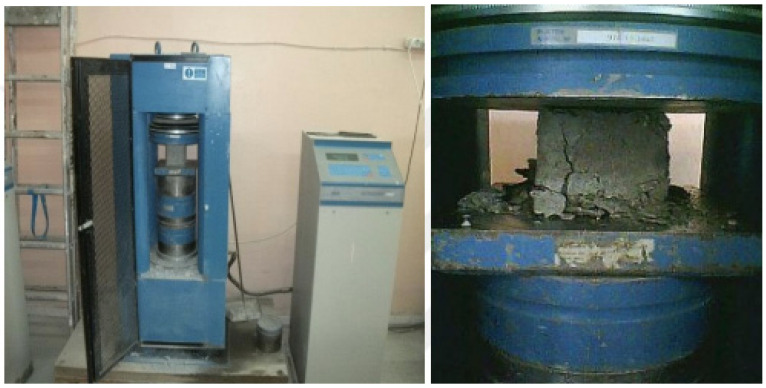
Compressive strength measuring device.

The compressive strength (*P_comp_*) tests were performed with an “Ele International” brand device according to TS 699 [30,31]. The “Ele International” brand device has a 3000 kN load capacity, a digital control panel, adjustable load speed, and the ability to apply force in a single axis. Figure 5 displays the measurement device.

**Figure 6:**
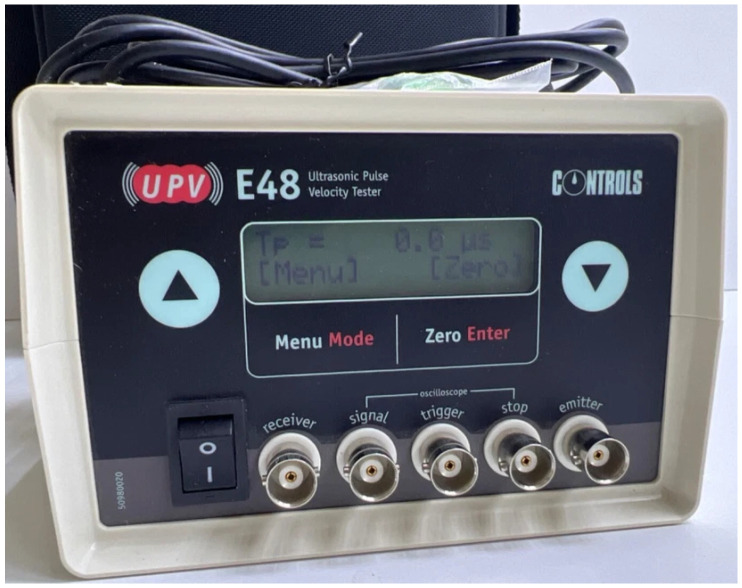
Ultrasonic pulse velocity (UPV) measuring device [32].

Ultrasonic pulse velocity (UPV) was measured using a Controls 58-E0048 model device. It conforms to the standards of EN 12504-4 and ASTM C597. The Controls 58-E0048 is a microprocessor-based UPV tester designed to evaluate the homogeneity, density, and structural integrity of concrete non-destructively. It operates with transducers typically ranging from 24 kHz to 150 kHz, and measures travel time from 0.1 μs to 1999.9 μs with a high resolution of 0.1 μs. Figure 6 shows the UPV measurement device [32].

The UPV measurements were made in two different directions from the surfaces of the cube-shaped samples in contact with the mold. The arithmetic means of the measurements were calculated. The device displayed the ultrasonic transit time in microseconds. The values were divided by the cube sample size to determine the ultrasound transmission rate.

Water absorption rate (WAR) measurements were performed in accordance with TSE 4045 [33] and BS 812-109 [34] standards, and WAR capacity was calculated as follows:

(2) $$WAR=100\left(\frac{m_{wet}-m_{dry}}{m_{dry}}\right)$$

where *m_dry_* is the dry mass of the sample (mass before starting the water absorption test), and *m_wet_* is the wet mass of each sample after being kept in a water tank for 24 h.

The density (*ρ*) of each sample is calculated by the ratio of the mass of the sample to the volume of the sample:

(3) $$\rho =\frac{m_{dry}}{V_{dry}}$$

where *V_dry_* is the volume of the dry sample in cm^3^. The dry mass *m_dry_* was measured using a precision digital balance with a maximum capacity of 500 g and a sensitivity of ±0.01 g.

## 3. Results and Discussions

### 3.1. Densities of the Samples

**Figure 7:**
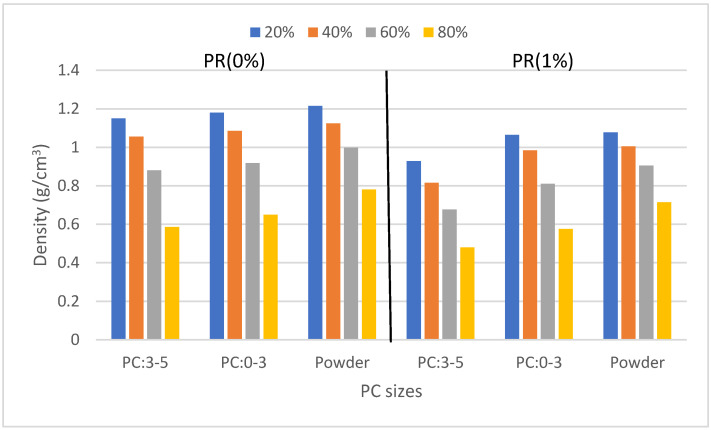
Measured densities of the samples.

Figure 7 displays the density of the newly produced samples. Adding PC particles into gypsum concrete changes the density mainly because you are replacing a dense mineral material with a lightweight, porous organic material—and that has several cascading effects. It should also be reported that PCs are lignocellulosic biomass, meaning they are made of cellulose, hemicellulose, and lignin. They are highly porous and are full of air voids. These properties result in lower densities compared to a traditional gypsum concrete.

From the figure, the density of the samples and the size of the PCs seem to be inversely related. The densities are measured as 0.170 g/cm^3^, 0.190 g/cm^3^, and 0.32 g/cm^3^ for the 3–5 mm, 0–3 mm, and powder form, respectively. Since larger particles generate larger voids and less mass, it is an expected result.

Additionally, the PR considerably reduces the density and raises total porosity. The resin swells and absorbs water during preparation; this moisture evaporates during curing, creating an artificial microporous network. The bulk density of the specimens is significantly reduced by the combined effect of these manufactured pores and the PC aggregates’ natural cellular structure.

Significant density reductions are observed when raising the PC content from 20% to 80%, as shown in Figure 7. In particular, in PR-free samples (0%), the densities of concretes with PCs in 3–5 mm, 0–3 mm, and powder forms decreased by 49.04%, 44.91%, and 35.72%, respectively. The reductions in PR-added samples are 48.27%, 46.00%, and 33.67%. In the PR-added samples with a 20% PC, the density decreases by 19.30%, 9.74%, and 11.27% for the PCs = 3–5 mm, PCs = 0–3 mm, and powder form, respectively. Density reduction solely attributable to PR addition are 18.08%, 11.53%, and 8.45% for samples with 80% PC content.

Compared to the co-author’s previous study [8], in which cement is used with PCs and PR instead of gypsum, the densities are lower. It is already expected since gypsum forms needle-like crystals that do not fully coat or densify plant particles. Also, gypsum has a higher water demand, leading to greater evaporation and, in turn, more pores in the concrete. The limited compaction retains internal voids from plant structure, and there is also a relatively weak bonding with plant fibers. This makes gypsum very lightweight. On the other hand, cement produces a C–S–H gel that coats and fills the gaps around plant particles. Lower residual porosity occurs compared to gypsum concretes. There is a stronger mechanical interlock (though still affected by sugars/extractives), which makes the cement heavier and stronger, yet lighter than normal concrete.

### 3.2. Thermal Conductivities of the Samples

**Figure 8:**
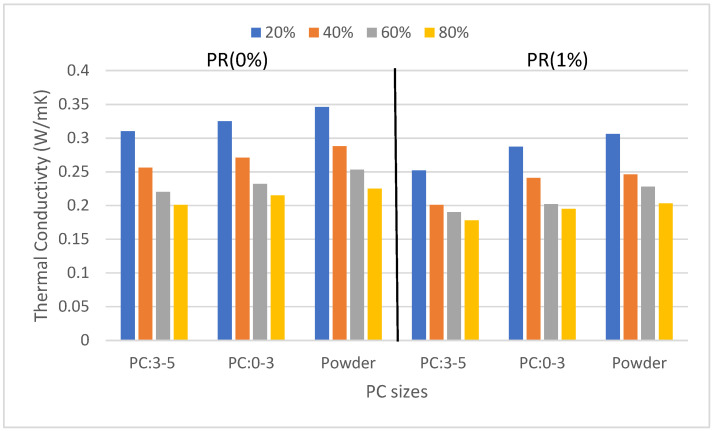
Measured thermal conductivities of the samples.

Figure 8 displays the measured thermal conductivities. Thermal conductivities decrease by 37.18% in PR-free samples with a particle size of 3–5 mm, 33.87% in samples with a particle size of 0–3 mm, and 34.97% in samples with the powder form of PCs. As the PC content in the samples increases from 20% to 80%, the decrements in samples with PR are 29.36%, 32.05%, and 33.66%, respectively. For samples with a 20% PC concentration, the specific decrease in thermal conductivity ascribed to PR addition is 18.70%, 11.69%, and 11.56%; for samples with an 80% PC content, it is 11.44%, 9.30%, and 9.77%.

This phenomenon occurs because, in addition to the PC porosity, a secondary network of artificial micropores forms during the 28-day curing period as water previously absorbed by the resin evaporates. Additionally, Figure 8 shows that samples with coarser particles (3–5 mm) have lower thermal conductivities than samples with finer particles (powder). This is explained by the fact that significant mechanical fragmentation reduces the internal porosity of individual PC particles, thereby increasing their bulk density and, consequently, the composite’s thermal conductivity.

The thermal conductivity of the composites with higher PC ratios and finer particles is similar to that of traditional perlite-aggregate gypsum plasters. Table 3 presents the comparison of thermal conductivities and densities with those in the literature. The PCs’ naturally porous structure and the PR’s modifying impact within the gypsum matrix are responsible for this advantageous thermal performance. As a direct result of the PR-induced artificial micropores, the PR-added samples showed significantly lower thermal conductivity values than the PR-free samples.

The values obtained here, when compared with the conductivity values reported in the authors’ previous studies [8], in which they mixed PCs and PR with cement to produce concrete, show that thermal conductivity is lower in the presence of gypsum. As aforementioned, using gypsum in the concrete results in very high porosity. There is poor packing of gypsum crystals, resulting in more voids in the concrete. However, the cement creates a denser, continuous matrix. The C–S–H gel bridges gaps reduce the air voids. Hence, there are more solid-to-solid heat conduction paths. This makes the PC-based concrete with cement a better thermal conductor than PC-based concrete with gypsum.

Compared with other plant-based concretes, PC samples exhibit lower, acceptable thermal conductivity [22,26]. However, as all tested samples exceed the ISO threshold of 0.065 W/mK [22] for heat-insulating materials, they are classified as lightweight concrete.

### 3.3. Compressive Strength of the Samples

**Figure 9:**
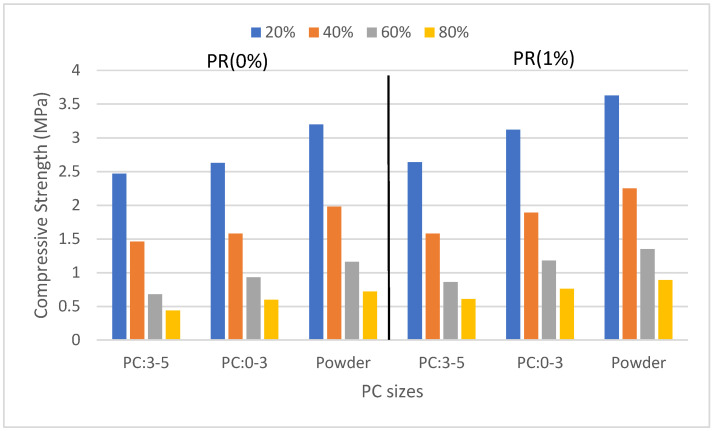
Measured compressive strengths of the samples.

Figure 9 shows the compressive strength test results. As the PC content increases from 20% to 80%, the samples’ compressive strength progressively decreases. The primary reason for this expected decline is the dispersion of the structural gypsum binder matrix by the very porous pebbles. The maximum compressive strength values of the PR-free samples in PCs = 3–5 mm, PCs = 0–3 mm, and powder form are 2.47 MPa, 2.63 MPa, and 3.20 MPa, respectively. In comparison, adding 1% PR to the samples increases the peak values to 2.64 MPa, 3.12 MPa, and 3.63 MPa.

Specifically, in the PR added samples, the compressive strength improves from 3.86% to 7.20% for the PCs = 3–5 mm, from 1.86% to 2.69% for the PCs = 0–3 mm, and from 1.34% to 4.83% for the powder form when comparing the 20% and 80% PC ratios. The PR’s curing and hardening during drying enhance the interfacial bonding properties of the gypsum matrix, leading to this mechanical enhancement. Furthermore, the research indicates that larger PC sizes have a more pronounced negative effect on overall compressive strength than finer aggregates.

It is known that the compressive strength of a typical gypsum plaster generally ranges from 2 MPa to 25 MPa [26]. This is heavily influenced by its type and water-to-powder ratio. Less porous, high-strength variants, such as dental stone, can exceed 30 MPa, whereas standard interior finish plasters typically achieve 2 to 5 MPa. In this context, the concretes with PC–PR–gypsum have lower values than a typical gypsum plaster without plant aggregate.

Compared with PC-based concrete with cement [8], the compressive strength values of the high-PC, PR-added samples were lower. Actually, compressive strength is dramatically higher in cement-based concretes. The gypsum has a weak matrix (gypsum crystals have limited cohesion compared to cement hydrates) and poor interfacial bonding with plant particles. Additionally, high porosity reduces the amount of load-bearing solid material. However, the cement-based concretes have a stronger binding phase because of the formation of the C–S–H gel. It leads to improved coating and encapsulation of plant particles. The lower porosity also leads to more effective load transfer and a stronger interfacial transition zone.

The results can also be compared to those of related research. For instance, by combining cement and coconut fiber with densities of 0.770 and 1.106 g/cm^3^, respectively, Khedari et al. [35] found compressive strengths of 1.97 and 2.53 MPa. Benazzouk et al. [36] produced samples containing rubber pieces and cement with densities of 0.625, 0.516, and 0.470 g/cm^3^. Those samples had compressive strengths of 23.30, 16.00, and 10.50 MPa. For samples of cement and wood chips with densities of 1.010, 0.870, and 0.700 g/cm^3^, respectively, Al Rim et al. [37] measured compressive strengths of 2.67, 2.35, and 1.35 MPa. These comparisons show that, although our results are not high enough, they are similar to the newly produced plant-based concretes in the literature.

### 3.4. Ultrasonic Pulse Velocity (UPV) of the Samples

**Figure 10:**
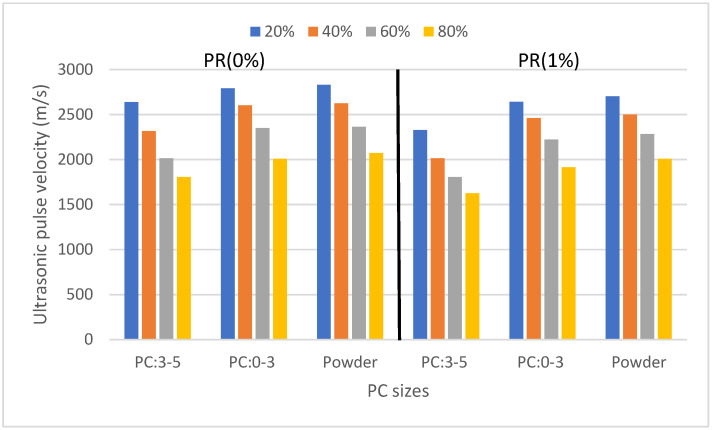
Measured utrasonic pulse velocities (UPVs) of the samples.

As anticipated, the samples’ density, compressive strength, thermal conductivity, and UPV all progressively decreased as the PC content in the mixtures increased (Figure 10). The UPV values for samples with 3–5 mm aggregates ranged from 1807 to 2640 m/s in PR-free samples and from 1625 to 2328 m/s in PR-added samples. For the PCs = 0–3 mm aggregate form, in PR-free and PR-added samples, the UPV values ranged from 2008 to 2793 m/s and 1916 to 2643 m/s, respectively. The structural correlations found during mechanical testing were also confirmed by the powder-form samples, which showed UPV ranges of 2073–2830 m/s for the control group and 2008–2703 m/s for the PR-added group.

Because of the high porosity, the air strongly attenuates ultrasonic waves. The discontinuous matrix (needle-like gypsum crystals), weak particle–matrix interface (scattering of waves), and presence of microcracks and voids result in low UPV values.

### 3.5. Water Absorption Rate (WAR) of the Samples

**Figure 11:**
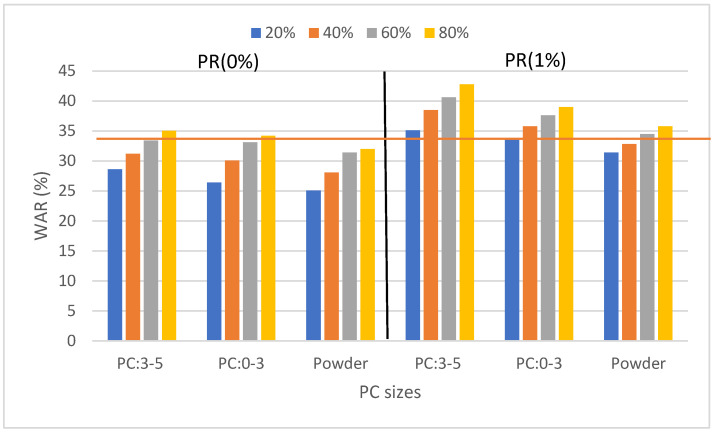
Measured water absorption rate (WAR) of the samples.

Figure 11 displays the WAR of the samples. The PC-aggregate gypsum plasters were found to have WAR values higher than 30%, especially when PR is added, as large-sized PC particles (3–5 mm) are used with a high weight ratio. For lightweight or bio-based concretes (e.g., with plant aggregates such as pine cones, hemp, or straw), the typical range of WAR is generally accepted to be 20–30% [24]. In the present study, some samples without PR can be assumed to be useful.

When the WAR values are compared to the values obtained for the PC-based concretes with cement [8], it is seen that the values are higher. There are some structural reasons for this result. First of all, the gypsum composites are hygroscopic. Second, high porosity causes a large capillary network, and the gypsum itself is water-sensitive and slightly soluble. Third, the PC aggregates absorb and retain water. Finally, the weak matrix allows easy penetration. Hence, adding PCs might have increased water absorption.

## 4. Conclusions

To provide a unique recycling approach for interior plastering and decorative applications, this study thoroughly assessed the value of waste PCs and PR as sustainable additives in gypsum-based composites. It is concluded that the thermal insulation properties of gypsum plasters are significantly improved by incorporating highly porous PC aggregates. In PR-added samples, the combined effect of the inherent PC cellular structure and the artificial micropores generated by the PR during curing substantially reduced both bulk density and thermal conductivity. Consequently, these novel composites are highly viable candidates for both thermal and potential acoustic insulation applications. Overall structural strength is inevitably reduced at higher PC ratios, although this loss was mitigated by adding 1% PR. These composite materials can be used for interior insulation and cosmetic purposes due to their high-water absorption; they should not be used in areas directly exposed to water. Ultimately, this research demonstrates that synergistically combining waste PC, PR, and gypsum yields a sustainable, lightweight, and thermo-mechanically efficient composite material for modern interior building applications.

## Figures and Tables

**Table 1:** Properties of the gypsum [28].

Physical form	White, powder
Standard consistency (water demand)	10 kg in 4–5 L of water
Initial time	130–180 min
Setting time	250–300 min
Dry density	900–1000 kg/m^3^
Compressive strength	≥2 N/mm^2^
Bending strength	≥1 N/mm^2^
Resistance to fire	A1
Standard	TS EN 13279-1,2

**Table 2:** Volumetric ratios and masses of the ingredients.

Code	Volumetric Ratio (%)			Mass (g)			
	PC	Gyp.	PR	PC	Gyp.	Total	PR
**PC: 3–5 mm**							
1	20	80	0	90	1520	1610	0
2	40	60	0	180	1140	1320	0
3	60	40	0	270	760	1030	0
4	80	20	0	360	380	740	0
**PC: 0–3 mm**							
5	20	80	0	120	1520	1640	0
6	40	60	0	240	1140	1380	0
7	60	40	0	360	760	1120	0
8	80	20	0	480	380	860	0
**Powder**							
9	20	80	0	180	1520	1700	0
10	40	60	0	360	1140	1500	0
11	60	40	0	540	760	1300	0
12	80	20	0	720	380	1100	0
**PC: 3–5 mm**							
13	20	80	1	90	1520	1610	16.10
14	40	60	1	180	1140	1320	13.20
15	80	20	1	360	380	740	0.74
16	80	20	1	360	380	740	0.74
**PC: 0–3 mm**							
17	20	80	1	120	1520	1640	16.40
18	40	60	1	240	1140	1380	13.80
19	60	40	1	360	760	1120	11.20
20	80	20	1	480	380	860	0.86
**Powder**							
21	20	80	1	180	1520	1700	17.0
22	40	60	1	360	1140	1500	15.0
23	60	40	1	540	760	1300	13.0
24	80	20	1	720	380	1100	11.0

**Table 3:** Measured thermal conductivities and densities of different materials [24].

Material	Density (g/cm^3^)	Thermal Conductivity (W/mK)
Exterior plaster	1.6	0.93
Interior plaster	1.8	1.163
Gypsum thin plaster (Perlite)	0.4–0.5	0.139–0.162
Gypsum rough plaster (Perlite)	0.4–0.5	0.139–0.162
Plaster with cement (Perlite)	0.7	0.244
Gypsum block (Perlite)	0.9	0.221

## Data Availability

The data presented in this study are available on request from the corresponding author.

## Nomenclature

*k*	Thermal conductivity of the sample (W/mK)
*m_dry_*	Dry mass of the sample (g)
*m_wet_*	Wet mass of sample after being kept in water (g)
*V_dry_*	Volume of the dry sample (cm^3^)
*ρ*	Density of sample (g/cm^3^)

## Abbreviations

PC	Pine cone
PR	Pine resin
WAR	Water absorption rate
UPV	Ultrasonic pulse velocity
